# Going digital – a commentary on the terminology used at the intersection of physical activity and digital health

**DOI:** 10.1186/s11556-022-00296-y

**Published:** 2022-07-16

**Authors:** Fabian Herold, Paula Theobald, Thomas Gronwald, Michael A. Rapp, Notger G. Müller

**Affiliations:** 1 Research Group Degenerative and Chronic Diseases, Movement, Faculty of Health Sciences, University of Potsdam, Karl-Liebknecht-Str. 24-25, 14476 Potsdam, Germany; 2 Institute of Interdisciplinary Exercise Science and Sports Medicine, MSH Medical School Hamburg, Am Kaiserkai 1, 20457 Hamburg, Germany; 3 Research Focus Cognitive Sciences, Division of Social and Preventive Medicine, University of Potsdam, Am Neuen Palais 10, 14469 Potsdam, Germany

**Keywords:** Digital Health, Electronic Health, Mobile Health, Telehealth, Telemedicine, Physical activity, Physical training, Aging

## Abstract

In recent years digital technologies have become a major means for providing health-related services and this trend was strongly reinforced by the current Coronavirus disease 2019 (COVID-19) pandemic. As it is well-known that regular physical activity has positive effects on individual physical and mental health and thus is an important prerequisite for healthy aging, digital technologies are also increasingly used to promote unstructured and structured forms of physical activity. However, in the course of this development, several terms (e.g., Digital Health, Electronic Health, Mobile Health, Telehealth, Telemedicine, and Telerehabilitation) have been introduced to refer to the application of digital technologies to provide health-related services such as physical interventions. Unfortunately, the above-mentioned terms are often used in several different ways, but also relatively interchangeably. Given that ambiguous terminology is a major source of difficulty in scientific communication which can impede the progress of theoretical and empirical research, this article aims to make the reader aware of the subtle differences between the relevant terms which are applied at the intersection of physical activity and Digital Health and to provide state-of-art definitions for them.

## Introduction

There is a substantial and still growing amount of evidence showing that regular physical activity (e.g., in the form of physical exercise and/or physical training – see Table 1 for definitions) is, among other lifestyle factors such as diet and sleep, an important factor to preserve or restore physical and mental health during the whole lifespan [14–18]. Although the above-mentioned evidence suggests that an adequate level of regular physical activity is a mandatory prerequisite to ensure overall health, worldwide more than two-thirds of adolescents [19] and almost one-third of adults [20–22] do not reach the recommended level of regular physical activity. From a public health perspective, the latter findings - indicating that a large amount of the world population has to be classified as physically inactive - are rather alarming [23]. In this context, there is also evidence that the level of physical inactivity increases as a function of age [22, 24, 25] and that older adults spend a relatively large amount of their wakening hours sedentary [26–29], whereby for the latter even an increase has been observed in the last years [28]. The fact that a considerable amount of the worldwide population does not reach the recommended level of physical activity [1, 2] has been significantly exacerbated because of the Coronavirus disease 2019 (COVID-19)-related public health actions (e.g., home confinement). Unfortunately, the latter has led in the general [30–36] and in the aging population [30, 33, 34, 37, 38] to a further increase in sedentary behavior and a decrease in the level of regular physical activity. Given that higher levels of physical inactivity and sedentary behavior are associated with detrimental health consequences [23, 39–43], the above-presented evidence suggests that, especially in the aging population, appropriate countermeasures such as interventions to promote regular and structured forms of physical activity - defined as physical exercise and/or physical training (see Table 1) - should be initiated. The latter idea is strongly reinforced by mounting evidence showing that regular and structured forms of physical activity are a crucial element of healthy aging [44–51].

**Table 1:** Overview of the definitions of physical activity, physical exercise, physical inactivity, physical intervention, physical training, and sedentary behavior. The definitions are based on the following literature [1–13]

Term	Definition
Physical activity	“...is defined as all muscle-induced bodily movements (e.g., in occupational or leisure time) leading to an increase in the energy expenditure above ∼1.0/1.5 MET (metabolic equivalent of the task; 1 MET = 1 kcal (4.184 kJ) • kg^− 1^ • h^− 1^).”
Physical exercise	“…is defined as a specific form of physical activity that is planned, structured, repetitive, and purposive to maintain or improve increasing or, at least, maintaining the performance in one or more fitness dimensions. Physical exercise can be differentiated based on temporal characteristics in acute (single bout/session of) physical exercise and chronic (multiple bouts/sessions of) physical exercise.”
Physical inactivity	“…is defined as an insufficient level of physical activity to meet specific recommendations (e.g., provided by the World Health Organization).”
Physical intervention	"...is an umbrella term that encompasses both physical exercise and physical training."
Physical training	“…is defined as chronic physical exercises being conducted regularly in a planned, structured, and purposive manner with the objective of increasing or, at least, maintaining the performance in one or more fitness dimensions.”
Sedentary behavior	“…is defined as behavior any waking behavior characterized by an energy expenditure of 1.5 METs or lower while sitting, reclining or lying.”

### Definition and delineation of the relevant terms

In recent years the utilization of digital technologies to promote regular and structured forms of physical activity has become a very popular field for both research and practical application, especially in the aging population [52, 53]. Indeed, there is some evidence that Electronic Health interventions [54] and Mobile Health interventions [55–57] (see next section for a definition and differentiation of the terms) can increase the regular level of physical activity in older adults.

**Fig. 1:**
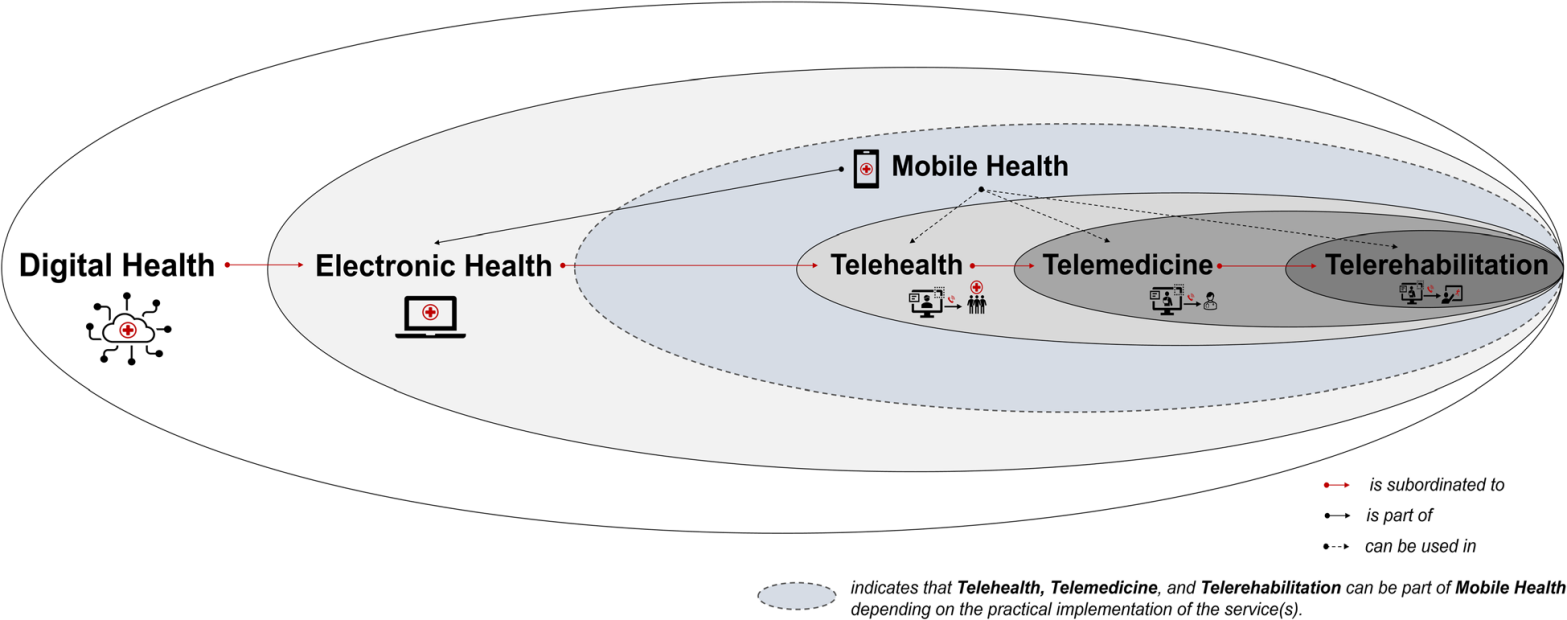
Schematic illustration of the relationships between Digital Health, Electronic Health (eHealth), Mobile Health (mHealth), Telehealth, Telemedicine, and Telerehabilitation

Several terms are commonly used when references are made to the application of digital technologies in different health care settings such as in the prevention and rehabilitation of (age-related) diseases. For instance, the terms (i) Digital Health, (ii) Electronic Health, (iii) Mobile Health, (iv) Telehealth, (v) Telemedicine, (vi) and Telerehabilitation are commonly used relatively interchangeably for the above-mentioned purpose as no generally accepted definition of these terms has yet been reached [58–79], which is probably caused by a distinct overlap in their meanings. As ambiguous terminology is a major source of difficulty in scientific communication which can impede progress in both theoretical and empirical research, we will at first provide definitions for (i) Digital Health, (ii) Electronic Health, (iii) Mobile Health, (iv) Telehealth, (v) Telemedicine, and (vi) Telerehabilitation (see also Fig. 1). Second, we discuss the implications of these definitions with regard to the promotion of physical activity.

- *Digital Health* is an umbrella term that covers the application of digital technologies in the context of health and, as shown in Fig. 1, is a subordinate construct that comprises both Electronic Health and Mobile Health [52]. According to the World Health Organization, Digital Health is rooted in Electronic Health, but also encompasses other related areas such as “big data”, genomics, and artificial intelligence [52].
- *Electronic Health (eHealth)* is, according to the World Health Organization, characterized by *“the use of information and communications technology (ICT) in support of health and health-related fields”* [52]. As a branch of Electronic Health, *Mobile Health (mHealth)* refers to *“the use of mobile wireless technologies for public health”* [52].
- Both *Telehealth* and *Telemedicine* encompass the utilization of electronic communications and information technologies to remotely provide health care services (e.g., when participants are at different locations) [80–82]. In particular, the term *Telehealth* compromises services of all health care professionals and thus also includes non-clinical services [80, 81]. In contrast, the term *Telemedicine*, in a narrow sense, refers specifically to clinical services [80, 82, 83]. As shown in Fig. 1, Telehealth and Telemedicine are both branches of Electronic Health [73, 79] and thus also a part of Digital Health [84].
- *Telerehabilitation* is a branch of Telemedicine and can be defined as the remote provision of rehabilitation services via telecommunication technologies [85–87]. As shown in Fig. 1, Telerehabilitation is a subordinate part of Telehealth, Electronic Health, and Digital Health.

Please note that Telemedicine and Telerehabilitation can be further subdivided [70, 88, 89], but discussing these subordinated branches is beyond the scope and aim of this article.

#### Implications with regard to physical activity

From a conceptual view, interventions aiming to promote physical activity through digital technologies fall within the scope of the definitions of Digital Health, Electronic Health, and Telehealth but do not necessarily fulfil the criteria of Mobile Health and Telemedicine. Since not all physical interventions that utilize digital technologies fall within the scope of Mobile Health and Telemedicine, the criteria used to characterize these theoretical constructs may constitute a valuable starting point for finding the appropriate wording.

1. With respect to Mobile Health, interventions aiming to promote physical activity through digital technologies are covered by the definition of Mobile Health when mobile and wireless technologies such as wearables and/or smartphones are used. Thus, when looking for an appropriate wording, the following questions should be answered: *Which digital technology has been used to deliver/receive the health-related intervention?*
2. Given the opinion that Telemedicine does refer specifically to remote clinical services [80, 82, 83], interventions aiming to promote, for instance, structured forms of physical activity such as physical exercise and/or physical training by utilizing digital technologies are, in a strict sense, only part of Telemedicine, when they are part of a clinical service. However, the latter interpretation strongly depends on the definition of Telemedicine. Based on the above-discussed aspects, answering the following question will help to identify the appropriate wording: *In which context has the health-related intervention been prescribed?*

### Practical examples - the devil is in the details

In this section, we will provide three examples to illustrate challenges in the attempt to derive an appropriate wording which in our view should be as narrow as possible and as broad as necessary.

The examples are based on the application of mobile apps that are delivered/received via smart devices such as smartphones and tablets, and videoconference software. Both applications have become popular tools to promote and deliver structured forms of physical activity [90, 91].

#### First example

There is an increasing number of mobile apps that are used to provide physical interventions in a non-clinical context to counteract the age-related decline of physical and cognitive capabilities [92–95]).

With reference to the first question of our proposed approach (*Which digital technology has been used to deliver/receive the health-related intervention?*) and based on the fact that the physical interventions in the first example rely on mobile apps (i.e., delivered/received via smartphone or tablet), these interventions fulfill the criteria of Digital Health, Electronic Health, Mobile Health, and Telehealth. However, as the apps are used in a non-clinical context (e.g., physical interventions to prevent the age-related decline of physical and cognitive capabilities [92–95]), they do not fall within the definitions of Telemedicine and Telerehabilitation. Thus, these interventions should be referred to as Telehealth and/or Mobile Health applications.

#### Second example

Comparable to the increase of mobile apps in the non-clinical context, there is also an increase of mobile apps for the use in clinical settings. For example, the Kaia app which is typically delivered/received via a mobile device (e.g., a smartphone) provides physical exercises for the therapy of (older) individuals suffering from back pain [96–98].

With respect to our proposed approach aiming to derive an appropriate wording, the answer to the first question *(Which digital technology has been used to deliver/receive the health-related intervention?*) is that smartphone apps fall within the definitions of Digital Health, Electronic Health, Mobile Health, and Telehealth, whereas they only meet the criteria for Telemedicine and Telerehabilitation when they are part of a clinical service (e.g., as a Digital Health application [DiGA]). The latter point is related to the second question (*In which context has the health-related intervention been prescribed?*). In this context, this mobile app can be referred to as a Telemedicine application, or in a narrower sense as a Telerehabilitation application because this mobile app is used to prescribe physical exercises in a clinical context (e.g., as therapy against back pain [96–98]).

#### Third example

There is a growing number of studies that use videoconference software in a non-clinical context to remotely deliver physical exercise sessions (i.e., online classes) to foster healthy aging [99–102].

As in the current example, the physical exercise sessions are delivered in a non-clinical context, these interventions do not fulfil the criteria of Telemedicine or Telerehabilitation (second question - *In which context has the health-related intervention been prescribed?*). Concerning the first question (*Which digital technology has been used to deliver/receive the health-related intervention?*) an explicit answer is rather difficult as it depends on the technological devices that are used to deliver/receive the content. In particular, when the online physical exercise sessions are solely delivered/received via stationary devices such as a television screen or a stationary computer, the definitions of Digital Health, Electronic Health, and Telehealth are met, but the criteria for Mobile Health are not fulfilled. In this case, Electronic Health and Telehealth can be used to refer to this intervention. However, when the same online content is solely delivered/received via a smartphone or a tablet, it fulfills the criteria of Digital Health, Electronic Health, Telehealth, and Mobile Health. In the latter case, we recommend, based on our approach, to refer to this intervention as a Mobile Health application. If both stationary (e.g., television screen) and mobile devices (e.g., smartphone) are used to receive the online physical exercise sessions, we suggest to refer to these interventions as Electronic Health or Telehealth applications since the criteria of Mobile Health are not met by each participant (e.g., only those who use a mobile device such as a smartphone to receive the intervention).

Based on the above-presented examples, it becomes apparent that an appropriate differentiation between Digital Health, Electronic Health, Mobile Health, Telehealth, Telemedicine, and Telerehabilitation can be very challenging as the definitions of these terms overlap to some extent. Moreover, although the approach proposed in this publication can serve as a guide to use an appropriate wording, it needs to be noted that a universal recommendation cannot be provided as the appropriate wording strongly depends on the particular case. In order to avoid misunderstandings, researchers should pay close attention to the subtle conceptual difference when they use the terms (i) Digital Health, (ii) Electronic Health, (iii) Mobile Health, (iv) Telehealth, (v) Telemedicine, and (vi) Telerehabilitation instead of using them relatively interchangeable. In addition, official bodies (e.g., World Health Organization) should make efforts to reach generally accepted definitions of the terms which are relevant in the field of physical activity and Digital Health.

## Conclusions

In recent years, digital technologies have become a popular tool for the promotion of physical activity (e.g., wearables, delivery of physical exercise sessions via online classes or smartphone apps) both in scientific research and in practical application [90, 91]. As terminological ambiguity can be a major source of difficulty impeding communication and thus progress in both theoretical and empirical research, here, we aimed to make the reader aware of the subtle differences between the relevant terms and provide state-of-the-art definitions for them. To that end, we hope that this article could provide the reader with a more nuanced view on relevant terms in the field of physical activity and Digital Health which, in turn, might foster further progress in this impactful research field.

## Data Availability

Not applicable.
